# Development of a 96-Well Plate Dithiothreitol Method for the Colorimetric Determination of Nickel Ions in Water Samples

**DOI:** 10.3390/s25175361

**Published:** 2025-08-29

**Authors:** George N. Nikas, Maria Tarara, George Z. Tsogas

**Affiliations:** Laboratory of Analytical Chemistry, School of Chemistry, Faculty of Sciences, Aristotle University of Thessaloniki, GR-54124 Thessaloniki, Greece; georgiosnn@chem.auth.gr (G.N.N.); mariatarara@chem.auth.gr (M.T.)

**Keywords:** colorimetric determination, nickel ions, dithiothreitol, smartphone detection, water samples

## Abstract

**Highlights:**

**What are the main findings?**

- A 96-well plate colorimetric method was developed for the quantification of Ni(II) with dithiothreitol (DTT).
- Sensitive determination of Ni(II) based on the colorimetric alteration of the Ni(II)-DTT reaction was achieved.

**What is the implication of the main finding?**

- A simple detection system, primarily based on a smartphone, was achieved.
- The proposed method was applied to surface water samples with sufficient sensitivity.

**Abstract:**

A new, simple, and accurate assay was developed for the colorimetric determination of nickel ions in 96-well plates. The proposed method utilized the color change immediately visible even to the naked eye during the reaction of Ni(II) ions with dithiothreitol molecules (DTT). The intensity of the color produced by the formation of the complex between nickel and dithiothreitol is linearly proportional to the concentration of the metal ions in an alkaline environment, at room temperature, and the detection was performed using a mobile phone as the detector. The proposed method had a good linear response between 0.05 and 0.75 mmol L^−1^ and a detection limit of 0.13 mmol L^−1^ and was successfully applied for the determination of Ni(II) in bottled and surface water samples.

## 1. Introduction

Nickel is a silvery-white, lustrous metal with a slight golden tinge, known for its strength, corrosion resistance, and high melting point. It is a transition metal that has an atomic number of 28, found primarily in minerals like pentlandite and garnierite [1,2]. One of its most common uses is in the production of stainless steel, where it enhances durability and resistance to oxidation. Nickel is also a key component in rechargeable batteries, including those used in electric vehicles, making it increasingly important in the shift toward sustainable energy solutions [3].

The analytical determination of nickel concentrations in environmental samples is important for assessing potential risks to human health, ecosystems, and water quality [4]. Nickel can enter the environment through industrial processes, mining, fossil fuel combustion, and waste disposal, and, at elevated levels, it can be toxic to both plants and animals [5]. Monitoring nickel helps identify pollution sources, ensures compliance with environmental regulations, and supports remediation efforts where contamination is detected [6]. It also provides critical data for evaluating the long-term impact of human activities on soil, water, and air quality, helping policymakers make informed decisions to protect public and environmental health [7].

Dithiothreitol (DTT) is an organic molecule commonly used as a reducing agent in biochemistry and molecular biology [8]. Its chemical formula is C_4_H_10_O_2_S_2_, and it contains two thiol (-SH) groups, which give it strong reducing properties. DTT is primarily used to break disulfide bonds within and between proteins [9]. DTT works by donating electrons to disulfide bonds (–S–S–), converting them into free thiol groups (–SH), which is essential when studying the structure or function of proteins under non-oxidizing conditions [10]. DTT can form complexes with metal ions like Ni(II) due to its two thiol (-SH) groups, which act as ligands that coordinate with metal centers [11]. These DTT-nickel complexes are of interest in coordination chemistry and bioinorganic studies because they mimic how sulfur-containing biomolecules (like cysteine or glutathione) interact with metal ions in biological systems [12].

Nickel can be determined in environmental, biological, and industrial samples using several analytical methods, each varying in sensitivity, specificity, and instrumentation, including Flame Atomic Absorption Spectrometry (FAAS) [13,14,15,16], Flow Injection Analysis–Flame Atomic Absorption Spectrometry (FIA-FAAS) [17], Graphite Furnace Atomic Absorption Spectrometry (GFAAS) [18,19,20,21], Inductively Coupled Plasma Atomic Emission Spectrometry (ICP-AES) [22,23,24], Inductively Coupled Plasma Mass Spectrometry (ICP-MS) [25], Adsorptive Stripping Voltammetry (AdSV) [26,27], Screen Printed Electrodes [28], X-ray Fluorescence (XRF) [29], and UV-Vis Spectrophotometry [30,31,32].

Although these techniques are accurate, have low detection limits, and can be successively applied to a varying number of samples, they require expensive instrumentation, costly consumables and reagents, and trained personnel for their correct handling to obtain accurate measurements. To address all of the above drawbacks, many analytical approaches have recently been developed for the colorimetric determination of metal ions using both paper micro-analytical devices and 96-well micro-plates, with measurements performed using electronic imaging devices such as mobile phones or flatbed scanners [33,34,35,36].

The most common Ni(II) colorimetric detection method is that with the use of dimethylglyoxime (DMG). The advantages of our method compared to the DMG method are simplicity and rapidity. In the DTT method, the addition of only two reagents is needed. The addition of the complexation reagent and the addition of the buffer solution. On the contrary, for the DMG method, there are two experimental paths. The gravimetric method and the colorimetric method. The gravimetric method is a method with excellent accuracy, but it is complex and difficult to apply, as it requires the quantitative formation of a precipitate (by adding concentrated ammonia), the filtration of this precipitate, which can take several hours, long drying in special kilns or ovens, and finally the weighing of the quantity of the precipitate. The colorimetric approach is also laborious because it is necessary to add bromine water to the nickel solution in order to ensure the oxidation of the divalent nickel ions to tetravalent. Then it is necessary to add concentrated ammonia, drop by drop, in order to destroy the excess bromine and decolorize the solution. Then a specific amount of absolute ethanol must be added, as the intensity and the stability of the color of the complex depend on its concentration. Finally, the DMG solution is added to form the characteristic orange color of the complex. Thus, the proposed method is faster, easier to implement, and with less waste produced.

In the present study, we report a new 96-well plate method for the determination of Ni(II) that relies on the colorimetric alteration after the complexation reaction between Ni(II) and DTT at mild alkaline pH. The analytical method proposed is convenient, rapid, without the requirement of technical expertise or laboratory instrumentation. The experimental procedure includes the sequential addition of the buffer solution for pH adjustment, the complexation reagent (DDT), and the analyte in the cells of the 96-well plate. The reaction was completed in less than 5 min at room temperature, a photograph of the 96-well plate cells was taken with a smartphone, and the ImageJ program (Version 1.53.k) was used for the measurement of the colorimetric intensity.

## 2. Materials and Methods

### 2.1. Chemicals and Solutions

**Figure 1:**
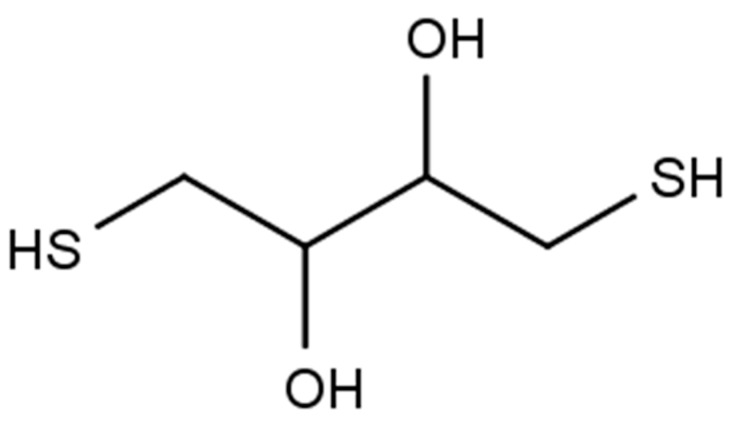
Structural formula of dithiothreitol (DTT).

All chemical substances were of analytical grade, and all the solutions were prepared with deionized water. DL-Dithiothreitol (DTT) solution 1 M in H_2_O (Figure 1) was provided by Sigma (St. Louis, MO, USA). Nickel sulfate hexahydrate (NiSO_4_ × 6H_2_O) and copper sulfate pentahydrate (CuSO_4_ × 5H_2_O) were purchased from Penta Chemicals Unlimited (Prague, Czech Republic). For the interference study, sodium chloride (NaCl), sodium hydrogen carbonate (NaHCO_3_), sodium sulfate anhydrous (Na_2_SO_4_), manganese chloride tetrahydrate (MnCl_2_ × 4H_2_O), zinc sulfate heptahydrate (ZnSO_4_ × 7H_2_O), lead nitrate (Pb(NO_3_)_2_), and chromium nitrate nonahydrate (Cr(NO_3_)_3_ × 9H_2_O) were supplied from Merck (Darmstadt, Germany). Additionally, calcium nitrate tetrahydrate (Ca(NO_3_)_2_ × 4H_2_O), potassium nitrate (KNO_3_), and iron nitrate nonahydrate (Fe(NO_3_)_3_ × 9H_2_O) were provided by Chem Lab NV (Zedelgem, Belgium). Moreover, sodium nitrate (NaNO_3_) and magnesium nitrate hexahydrate (Mg(NO_3_)_2_ × 6H_2_O) were bought from Panreac Quimica SA (Barcelona, Spain). Finally, cobaltous nitrate hexahydrate (Co(NO_3_)_2_ × 6H_2_O) and cadmium nitrate tetrahydrate (Cd(NO_3_)_2_ × 4H_2_O) were provided by Sigma (St. Louis, MO, USA). Different cation and anion stock solutions were prepared for the selectivity study in deionized water. Finally, sodium nitrate stock solution (2.0 mol L^−^^1^), used for the salinity study, was prepared by dissolving the appropriate amount in deionized water.

For the interference study, alkali metal (Na^+^, K^+^) stock solutions of 200 and 100 mg L^−1^ were prepared by the appropriate dilution, respectively. For the alkaline metal earth solutions (Ca^2+^, Mg^2+^), stock solutions of 400 mg L^−1^ were prepared by the appropriate dilution. For the anion interference study (NO_3_^−^, SO_4_^2−^, HCO_3_^−^, Cl^−^), the production of stock solutions of 200, 100, 2000, and 400 mg L^−1^, respectively, was performed by the appropriate dilution of the aforementioned salts in deionized water. Finally, the possible interference by transition metal elements was achieved by the production of stock solutions of 200 mg L^−1^ in deionized water.

### 2.2. Apparatus

The pH measurements to optimize the influence of the pH in this method were carried out using a pH meter (CONSORT, Turnhout, Belgium). The experiments were conducted in white sterile 96-well plates with a transparent bottom (BRAND GMBH, Wertheim, Germany). The UV-Vis measurements were performed using a JASCO V-530 UV/Vis spectrophotometer and matched quartz cells of 1 cm path length. The photographs of the micro-plates were taken from the same distance, angle, and maintaining the same lighting conditions, using as an imaging device a smartphone (Motorola 50 Edge Neo, Merchandise Mart, Chicago, IL, USA). The camera of the mobile phone has the following characteristics: triple camera with 50 MP, f/1.8, 24 mm (wide), 1/1.55″, 1.0 µm, multi-directional PDAF, OIS; 10 MP, f/2.0, 73 mm (telephoto), 1/3.94″, 1.0 µm, PDAF, OIS; and 3x optical zoom 13 MP, f/2.2, 13 mm, 120° (ultrawide), 1/3.0″, 1.12 µm, PDAF.

### 2.3. Experimental Procedure

**Figure 2:**
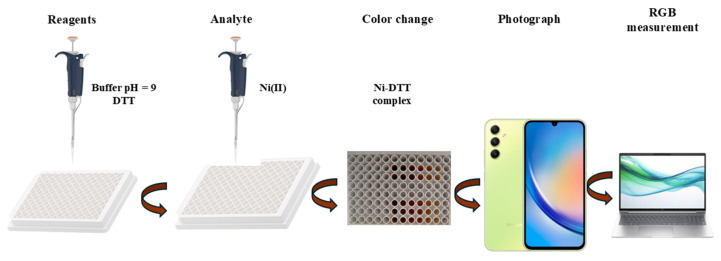
Schematic illustration of the experimental procedure.

The experimental protocol applied to the proposed analytical process was based on the lack of need for laboratory equipment and specialized personnel and briefly included the addition to each cell of small volumes of (a) the buffer solution for pH regulation (pH = 9), (b) the complexing reagent (DTT, 2.0 µL of 1.0 mol L^−1^), and (c) the analyte (Νι^2+^), to a final volume of 300 μL, by the addition of deionized water (Figure 2).

Deionized water was used instead of Ni(II) for the blank samples. The complexation reaction was completed in less than 5 min at room temperature, and the photograph of the 96-well plate cells was taken via a smartphone camera and successively saved as a JPEG file. The measurement of the color intensity was carried out with the ImageJ program by selecting the same area in every measurement, using the round shape tool of the program. Then, the RGB mode was applied to the colored and the red area of the RGB stack of the program. The variation of the color intensity was measured, and the results were passed on to an Excel sheet.

### 2.4. Comparison UV-Vis Method

With the UV-Vis method, a reference calibration curve was achieved with standard nickel ion concentrations ranging from 1.0 to 20 mg L^−1^. From the stock Ni(II) solutions, 1.0 mL was taken and placed in the cuvette, and, with a micropipette, 1.0 mL of the 10 mM phosphate buffer solution, and then 1.0 mL of the 1.0 mM DTT solution, were added. The cuvette was left for 5 min at room temperature, under dark conditions, for the complex to be formed and measured at 465 nm for each concentration [11]. The same experimental procedure was followed for measuring the unknown samples.

### 2.5. Real Samples

The proposed method was tested on ten natural water samples, including tap water, bottled water, seawater, and river water. Six bottled water samples were obtained from local stores in Thessaloniki. Two river water samples and a seawater sample were collected in 500 mL glass containers. River water samples were gathered from the Acheron and Louros Rivers located in northwestern Greece, in the region of Epirus, and the seawater sample was taken from Thermaikos Gulf (near the city of Nea Moudania). All samples were filtered to remove algae and suspended particles with a paper filter (Whatman 0.45 μm, Merck, Darmstadt, Germany). The tap water sample was collected from the supply network of the city of Thessaloniki. All samples were protected from light at 4 °C until analysis. The application of this method directly to industrial wastewater samples would be difficult to successfully carry out due to the complexity of the sample, possible substances that would hinder the detection of nickel, and its low concentrations. However, in recent years, many techniques have been developed for the removal of nickel ions from such samples by adsorption on materials such as activated carbon in its various forms, zeolites, and nanomaterials, which could be used to create aqueous extracts that could be measured by this method.

## 3. Results and Discussion

### 3.1. Effect of pH

Nickel(II) tends to form coordination complexes with sulfur donors, particularly thiol groups [37], while DTT can act as a bidentate ligand, coordinating to nickel via both thiol groups [11]. The resulting complex is typically a square planar or octahedral geometry, depending on the number of ligands and the presence of other coordinating species [11].

**Figure 3:**
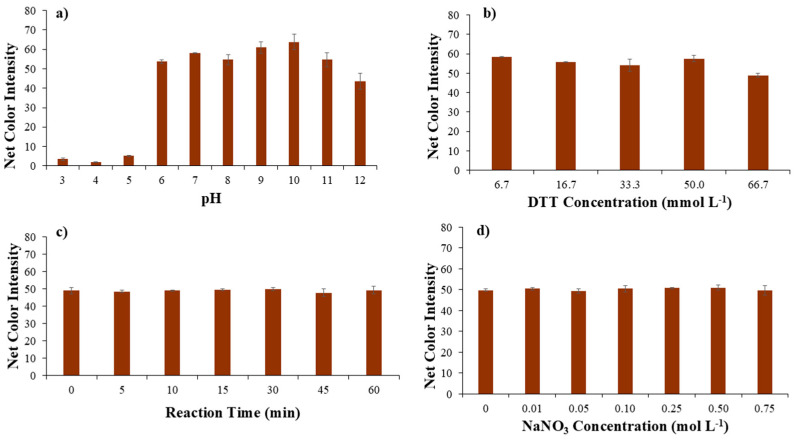
Optimization of (**a**) pH value, (**b**) DTT concentration, (**c**) reaction time, and (**d**) ionic strength as NaNO_3_ concentration affecting the performance of the proposed method.

Thus, pH has a significant role in the binding ability of DTT to Ni(II). In strongly acidic conditions (pH < 5), thiol groups in DTT are protonated, thus less nucleophilic, exhibiting weak binding to Ni(II), while, in mild pH conditions (pH from 6 to 8), thiol groups are partially deprotonated, and DTT can bind Ni(II) effectively, and, finally, in alkaline conditions (pH > 8), thiol groups are fully deprotonated, with strong binding to Ni(II) [38]. Our findings are in complete agreement with the literature, as for acidic pH values; as is evident from Figure 3a, the color change due to the formation of the complex is minimal.

For neutral and mild alkaline pH values, the color change increases significantly, which indicates the complete formation of the Ni-DTT complex, but, for higher alkaline values (pH > 10), a significant decrease was observed, which comes in an agreement with previous findings that reported oxidation of DTT and, as a sequence, decrease in the color intensity [39]. DTT half-life was estimated to be 1.4 h at pH = 8.5 at room temperature, with an additional decrease as the pH value increases [40]. Therefore, a pH value equal to 9 was used in the following experiments.

### 3.2. Effect of DTT Concentration

The concentration of DTT plays a significant role in the formation and stability of nickel–DTT complexes. When the concentration of DTT is low relative to Ni(II), only a small fraction of these ions will bind to DTT, resulting in monodentate or weak bidentate complexes. On the other hand, at very high DTT concentrations, oxidation of DTT (to its disulfide form, DTTox) becomes a concern, particularly in air or over prolonged exposure. Thus, the second parameter studied was the DTT concentration. As is evident from Figure 3b, the concentration of DTT has a minor effect on the development of the Ni-DTT complex and consequently on the formation of the color. Only at the highest concentration is a small decrease observed, which may be due to the oxidation of DTT, which is in agreement with previous studies [39]. Consequently, the lowest DTT concentration was used as the optimal one to save reagents and reduce waste.

### 3.3. Effect of Reaction Time

Another parameter studied was the duration of the complexation reaction between Ni(II) and DTT. The influence of the reaction time was studied in the range of 0 to 60 min with different time intervals (Figure 3c). The complexation reaction of Ni(II) with DTT was not favored over time, as the color intensity measured was similar for all the periods studied, making the complex formation very stable. Thus, a time of 1 min was chosen for taking the photo, making the proposed method extremely fast.

### 3.4. Effect of Ionic Strength

Ionic strength impacts the formation, stability of complexes, and robustness of analytical methods several times, regarding the spectrophotometric assessment of the Ni(III)-DTT complex [11]. Thus, in this assay, the effect of ionic strength was considered important and was studied for a range of sodium nitrate concentrations. Different concentrations of NaNO_3_ solutions (ranging from 0.01 to 0.75 mol L^−1^), obtained after dilution from the stock solution of 2.0 mol L^−1^, were added into the micro-plate’s wells. As a result, no significant color change was observed, and, from Figure 3d, it is clear that the ionic strength did not affect the proposed methodology. Consequently, no NaNO_3_ addition was selected for the next experiments.

### 3.5. Method Validation

In analytical chemistry, the validation of the assay is an important process to ensure that a proposed method is suitable for its purpose. It involves a systematic evaluation of many performance characteristics of the assay, including accuracy, precision, selectivity, linear range, detection limit, and quantitation limit, under specific principles.

#### 3.5.1. Selectivity

**Figure 4:**
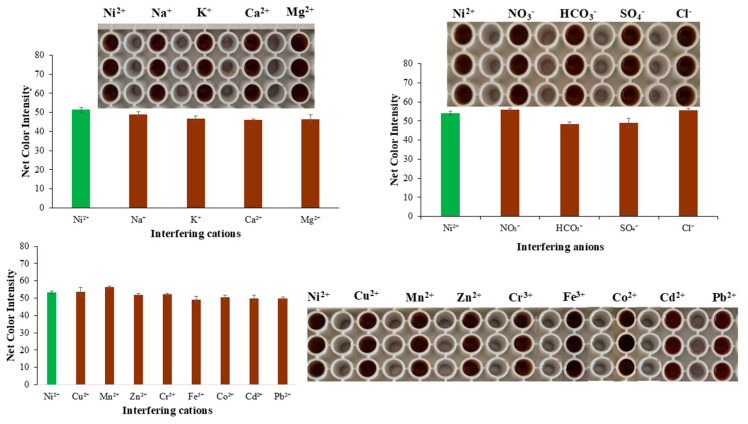
Interference study of the proposed method.

The selectivity of the assay was validated by the determination of ions present in water samples, which possibly hinder the formation of the complex we were studying. Additionally, specific cations that have similar physicochemical properties to Ni(II) were studied to find possible interferences during the complex formation and the performance of the proposed method (Figure 4).

The cations and anions that are most abundant in water samples are Na^+^, Ca^2+^, Mg^2+^, K^+^, NO_3_^−^, HCO_3_^−^, SO_4_^2−^, and Cl^−^. Thus, with appropriate dilutions from stock ion solutions, the working ion solutions were produced with concentrations commonly found in water samples (Table 1), and Ni(II) was at a concentration of 5.0 mg L^−1^. The only cation that can interfere in the determination of Ni(II) is Fe(III). To answer this drawback, the complexation of Fe(III) with fluoride ions can be selected. Fluoride ions, when added to a solution containing both iron(III) and nickel(II) ions, will preferentially bind to the iron(III) ions, forming the complex ion [FeF_6_]^3−^. This complex is stable and prevents the iron(III) ions from interfering with subsequent reactions or analysis. Thus, the addition of F^−^ in excess can fully mask iron(III), and no color formation would be expected.

#### 3.5.2. Figures of Merit

**Figure 5:**
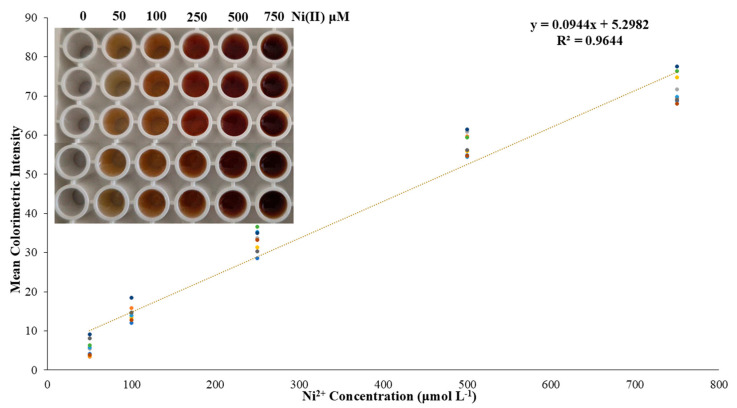
The accumulated calibration curve of the proposed method. Enclosed is a typical photograph of the color alteration because of different Ni(II) concentrations.

After the determination of the optimum conditions for metal and DTT complexation, as well as studying the selectivity, it was decided to create a series of calibration curves to design a cumulative calibration curve that would consist of many measurements on many different days of conducting the experiments. So, experiments were performed involving five different Ni(II) concentrations, namely, 0.05–0.10–0.25–0.50–0.75 mmol L^−1^, which is the linear range of the proposed method, since for higher Ni(II) concentrations the color reached a maximum, and the calibration curve reached a plateau. The method can be optimized to improve sensitivity by (a) the use of nanomaterials including AuNPs or AgNPs to induce the color change and enhance the sensitivity of the method; (b) the use of preconcentration techniques including solid-phase extraction (SPE) or liquid–liquid extraction (LLE) to separate and concentrate Ni(II) from the sample matrix before the colorimetric detection, which can significantly improve sensitivity, especially for low concentrations; (c) controlling ambient light interference with a stable light source, using a uniform and stable light source, like an LCD display, as a background for image acquisition to minimize ambient light fluctuations; (d) enclosing the reaction 96-well plate and the smartphone camera within a light-proof chamber that shields the sample from external light sources; and (e) going beyond basic RGB analysis, exploring different color spaces (HSV, CIELab) and novel channel combinations that can significantly improve sensitivity and reduce inter-phone variations during the color changes. Three calibration curves were performed each day, for three consecutive days, providing, through this analytical proposed protocol, a total of nine different calibration curves, whereby a total of 45 measurements of nickel concentrations were determined. The average colorimetric intensity measurements of the three days are shown in the accumulated calibration curve presented in Figure 5.

The intra-day precision for 0.25 and 0.50 mmol L^−1^ was found as the relative standard deviation (RSD) values, which were 4.0 and 4.3% for the aforementioned Ni(II) concentrations, respectively, while the inter-day precision was 7.8 and 4.9% for the same concentrations. Finally, the limits of detection and quantification (LOD-LOQ)were calculated by the standard deviation of the regression’s equation constant term and the slope provided from the calibration curve as LOD = 3.3 × SD/s and LOQ = 10 × SD/s, where SD is the standard deviation of the blank sample, and s is the slope of the regression equation. Thus, the LOD of this assay was calculated to be 0.13 mmol L^−1^, and the LOQ was 0.40 mmol L^−1^. The sensitivity of the method is similar to that of flame atomic absorption spectrometry. The utilization of a preconcentration method or a standard addition method can significantly improve the applicability of the proposed method in such samples. Additionally, a dark chamber with certain lighting with a stable camera position could improve the analysis of the photographs taken, as well as the usage of a smartphone with higher camera characteristics.

### 3.6. Application of Real Samples

The applicability of the proposed method was determined for ten different surface water samples, including six bottled water samples, two river water samples collected from two different rivers of Epirus region in northwestern Greece, one seawater sample collected from the seaside of Nea Moudania, and, finally, a tap water sample collected from the water supply network of the Aristotle University of Thessaloniki campus. The samples were handled as aforementioned in the Section 2, and, as initially no Ni(II) was determined in all of the samples, the standard addition method was implemented, and two known concentrations of Ni(II) were added to each water sample, and the results are shown in Table 2. The recoveries of the measured spiked levels of Ni(II) were satisfactory and between 82.9 and 104.9%.

### 3.7. Comparison with UV-Vis Method

The accuracy of the proposed colorimetric method was estimated with the measurement of the same real water samples with a UV-Vis method. After the addition of specific amounts of Ni(II) in bottled water samples, a measurement of each sample with both methods was accomplished. As is evident from Table 3, the recoveries between the two methods were between 93 and 108%, proving the accuracy of the method.

## 4. Conclusions

An instrument-free analytical method has been developed and validated for the direct determination of Ni(II) in surface water samples. Quantification of the analyte was performed by measuring the linear growth of the color intensity with the Ni(II)-DTT complex produced by the analyte. The assay developed was fast, simple, and accurate, while it performed good selectivity against a variety of possible interfering substances. The advantages of our method compared to the most established colorimetric method (DMG method) are the simplicity and the rapidity. In the proposed DTT method, the addition of only two reagents is needed, the addition of the complexation reagent and the addition of the buffer solution. The suggested methodology can be easily followed by non-specialized personnel, including undergraduate students, using very small volumes of reagents and in a very short period. The proposed method can be used as a turn-on/off switcher, indicating the existence of Ni(II) or for the analytical determination of its concentration. The flowback of complexing DTT with trivalent iron and cobalt ions can be easily eliminated by masking with fluoride ions. Conclusively, the method presented acceptable detection limits (0.13 mmol L^−1^) with good applicability in tap, sea, river, and bottled water samples, with % recoveries ranging from 83.0 to 105.1%.

## Figures and Tables

**Table 1:** Concentration of the possible interfering ions used for the selectivity study.

Ions	Concentration (mg L^−1^)	Ions	Concentration (mg L^−1^)
Na^+^	50	NO_3_^−^	50
Ca^2+^	100	HCO_3_^−^	250
Mg^2+^	50	SO_4_^2−^	25
K^+^	25	Cl^−^	50
Cu^2+^	25	Mn^2+^	25
Zn^2+^	25	Co^2+^	5.0
Fe^3+^	5.0	Cr^3+^	10
Pb^2+^	25	Cd^2+^	25

**Table 2:** Applicability of the assay in surface water samples.

Water Samples	Spiked (mg L^−1^)	Found (mg L^−1^)	Recovery (±Standard Deviation, %, n = 3)
Bottled 1	5.87 29.3	5.63 27.9	95.9 ± 5.3 95.2 ± 4.1
Bottled 2	5.87 29.3	5.05 27.1	86.0 ± 4.0 92.5 ± 6.6
Bottled 3	5.87 29.3	4.99 26.1	85.0 ± 3.5 89.1 ± 6.0
Bottled 4	5.87 29.3	5.46 27.1	93.0 ± 5.5 92.5 ± 6.1
Bottled 5	5.87 29.3	4.87 29.1	83.0 ± 6.5 99.3 ± 2.1
Bottled 6	5.87 29.3	5.05 30.2	86.0 ± 3.8 103.1 ± 6.5
River 1	5.87 29.3	5.52 26.3	94.0 ± 3.5 89.8 ± 3.1
River 2	5.87 29.3	5.58 25.8	95.1 ± 7.4 88.0 ± 2.1
Sea	5.87 29.3	4.98 26.5	84.8 ± 0.7 90.4 ± 7.0
Tap	5.87 29.3	5.12 30.8	87.2 ± 3.2 105.1 ± 6.0

**Table 3:** Comparison of the proposed assay with a UV-Vis method.

Samples	Added (mg L^−1^)	Proposed Method (mg L^−1^)	UV-Vis Method (mg L^−1^)	% Recovery (Value Found/Value Added × 100%)	% Recovery (Value Proposed Method/Value UV-Vis × 100%)
1	8.0	8.4	8.8	105.0	94.9
2	8.0	8.0	8.5	100.0	93.6
3	8.0	8.2	8.7	102.5	94.3
4	9.5	9.9	9.2	104.2	108.0
5	9.5	9.5	9.1	100.0	104.5
6	9.5	9.4	9.0	98.9	104.2

## Data Availability

The original contributions presented in this study are included in the article.

## Abbreviations

The following abbreviations are used in this manuscript:

DTT	Dithiothreitol
FAAS	Flame Atomic Absorption Spectrometry
FIA-FAAS	Flow Injection Analysis–Flame Atomic Absorption Spectrometry
GFAAS	Graphite Furnace Atomic Absorption Spectrometry
ICP-AES	Inductively Coupled Plasma Atomic Emission Spectrometry
ICP-MS	Inductively Coupled Plasma Mass Spectrometry
AdSV	Adsorptive Stripping Voltammetry
XRF	X-ray Fluorescence
UV-Vis	Ultra Violet–Visible
JPEG	Joint Photographic Experts Group
RGB	Red Green Blue
